# Supplementary material for: The conservation of human functional variants and their effects across livestock species
Source: Commun Biol. 2022 Sep 21;5:1003. doi: 10.1038/s42003-022-03961-1 (PMC9492664; doi:10.1038/s42003-022-03961-1)
Supplement: Supplementary file 2 — Supplementary Information [file 42003_2022_3961_MOESM2_ESM.pdf]

**Supplementary Information**

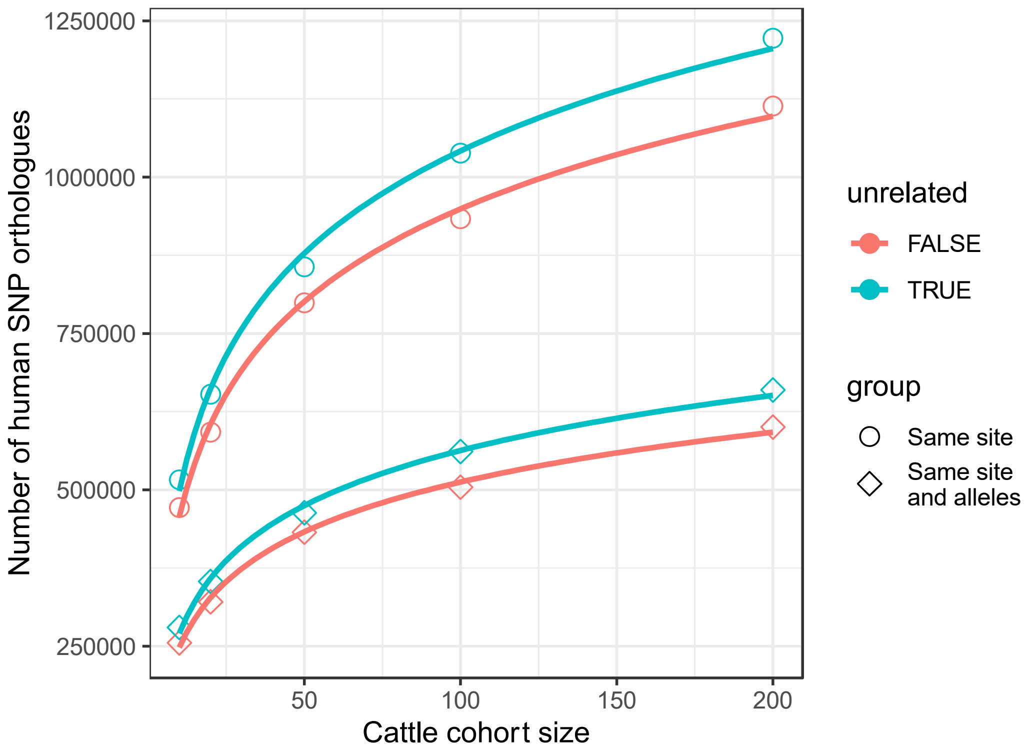


Supplementary Figure 1. The effect of cohort relatedness on the number of orthologues of human SNPs found in cattle. The red line shows the number of orthologues found when no filtering based on relatedness was applied to the cohort. The cyan lines show the effect of excluding from the cohort related animals so that all those remaining have a kinship coefficient[1] with each other equal to or less than 0.

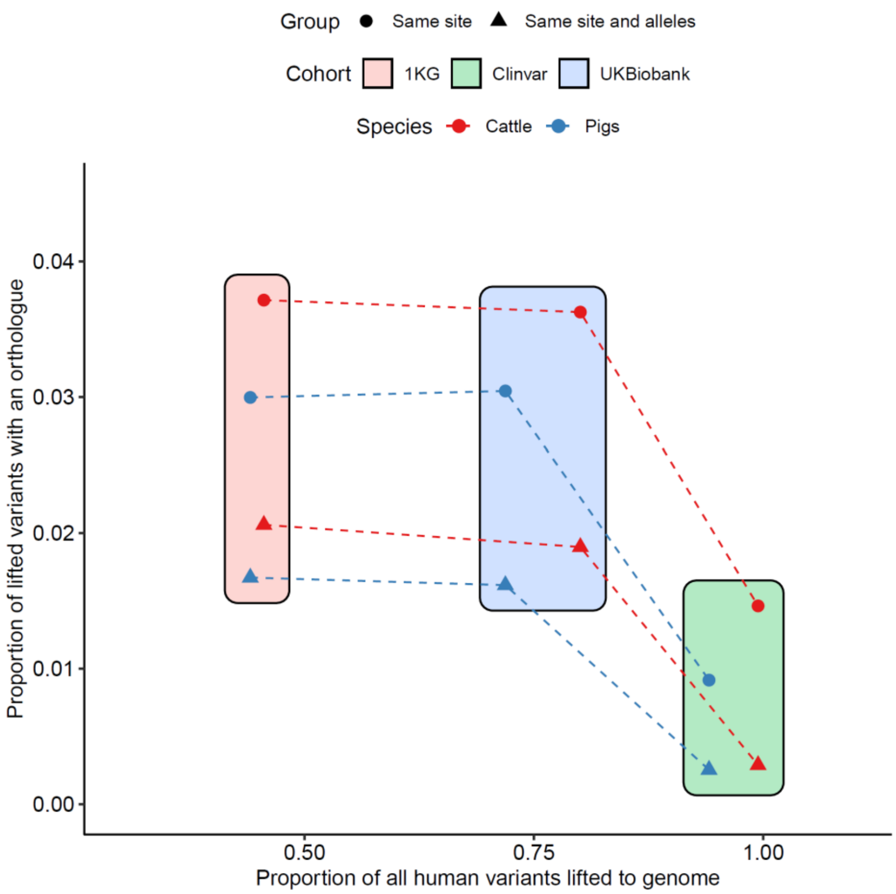


Supplementary Figure 2. The proportion of human variants that have a livestock orthologue by variant set. Although most Clinvar variants could be lifted to a position in the cow and pig genomes, fewer than expected had orthologues either with or without the same alleles. This likely reflects the strong selection against these changes.

## Supplementary References

1. Manichaikul, A. *et al.* Robust relationship inference in genome-wide association studies. *Bioinformatics* **26**, 2867–2873 (2010).
