# Supplementary material for: The conservation of human functional variants and their effects across livestock species
Source: Commun Biol. 2022 Sep 21;5:1003. doi: 10.1038/s42003-022-03961-1 (PMC9492664; doi:10.1038/s42003-022-03961-1)
Supplement: Supplementary file 3 — Description of Additional Supplementary Files [file 42003_2022_3961_MOESM3_ESM.pdf]

# Description of Additional Supplementary Files

**File name:** Supplementary Data 1
**Description:** The source data behind Figure 1.

**File name:** Supplementary Data 2
**Description:** The source data behind Figure 2a.

**File name:** Supplementary Data 3
**Description:** The source data behind Figure 4.

**File name:** Supplementary Data 4
**Description:** Clinvar variants linked to different phenotypes found across species.

**File name:** Supplementary Data 5
**Description:** The source data behind Figure 5.

**File name:** Supplementary Data 6
**Description:** The source data behind Figure 6.

**File name:** Supplementary Data 7
**Description:** The source data behind Figure 7.

**File name:** Supplementary Data 8
**Description:** The source data behind Figure 8.
